# Dataset of organic sample near infrared spectra acquired on different spectrometers

**DOI:** 10.1016/j.dib.2020.106264

**Published:** 2020-09-02

**Authors:** Céline Chauvergne, Laurent Bonnal, Denis Bastianelli, Hélène Carrère, Yves Griveau, Marie-Pierre Jacquemot, Matthieu Reymond, Valérie Méchin, Virginie Rossard, Éric Latrille

**Affiliations:** a INRAE, Montpellier University, LBE 102 Avenue des Étangs, 11100 Narbonne, France; b CIRAD, UMR SELMET, F-34398 Montpellier, France; c Institut Agro, SELMET, University of Montpellier, CIRAD, INRAE, Montpellier, France; d Institut Jean-Pierre Bourgin, INRAE, AgroParisTech, Université Paris-Saclay, 78000 Versailles, France

**Keywords:** Near infrared spectroscopy, Spectrum transfer model, Chemometrics, Organic samples

## Abstract

This dataset presents 127 raw near infrared spectra of different organic samples acquired on three different spectrometers in three different labs. An example of data processing is shown to create six spectra transfer models between the three spectrometers (two by two). In order to build and validate these transfer models, the dataset was split into two sets of spectra: a first set was used to compute six spectra transfer models thanks to the Piecewise Direct standardisation function (PDS). A second set of spectra, independent of the first one was used to validate transfer models. Spectrum treatments and models were created on ChemFlow (https://vm-chemflow-francegrille.eu/), a free online chemometric software that includes all the necessary functions.

## Specifications Table

**Table:** 

Subject	Spectroscopy
Specific subject area	Near infrared spectroscopy, chemometrics
Type of data	Table Figure
How data were acquired	Sample spectra were acquired using three different near infrared spectrometers: NIRFlex N-500 FT-NIR (BUCHI, France, serial number 1,000,001,305) NIR-System5000 (FOSS, Denmark, serial number 94,630,632) ANTARIS II FT-NIR (Thermo Fisher Scientific, USA, serial number AHY0800337)
Data format	Raw
Parameters for data collection	Near infrared spectra of various organic samples were acquired on the three spectrometers in the standard using conditions. Samples were analysed in either vials or cups.
Description of data collection	Organic samples were dried and ground (<1 mm) before acquiring near infrared spectra.
Data source location	LBE, INRAE, Narbonne, France SELMET, CIRAD, Montpellier, France IJPB, INRAE, Versailles, France
Data accessibility	Data are available on public repositories: Repository name: Data INRAE (https://data.inrae.fr/) Data identification number: doi:10.15454/XFTLV4 Direct URL to data: https://doi.org/10.15454/XFTLV4 Repository name: Zenodo Data identification number: doi:10.15454/XFTLV4 Direct URL to data: https://zenodo.org/record/3908210#.Xvmx_igzZPY

## Value of the Data

• These data can be used as supplements for the description of organic matter samples and can be compared to other studies.
• Spectra were acquired using three specific NIR spectrometers and they could be compared to spectra provided by other devices. Any researcher can use these data to perform chemometric analyses.
• This dataset shows the value of near infrared spectroscopy coupled with chemometrics for inter-laboratory works.

## 1 Data Description

Data provided in this article are 127 raw near infrared spectra of different organic samples acquired on three different spectrometers in three different labs.

**Fig. 1:**
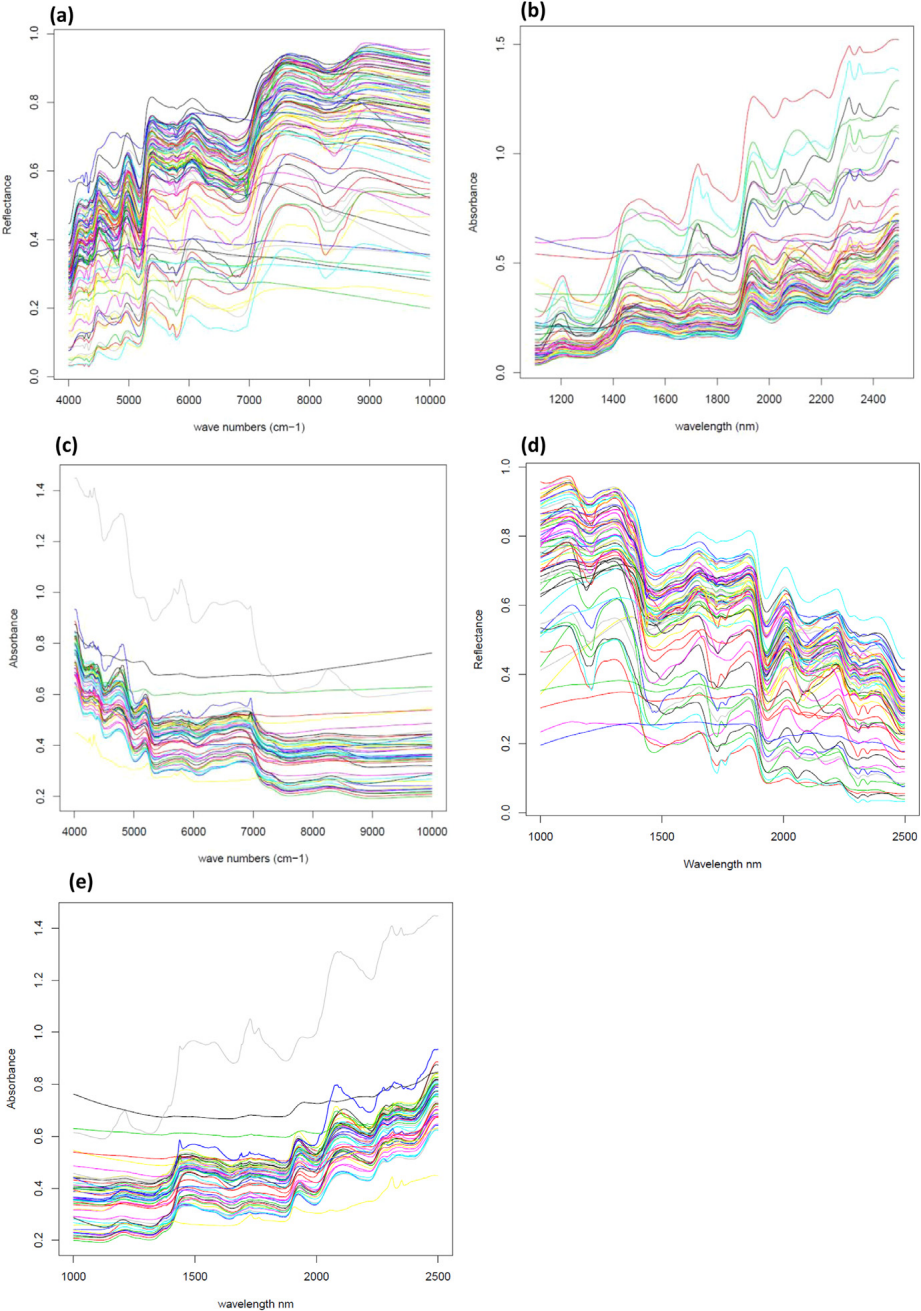
**a.** 92 averaged BUCHI NIRFlex N-500 spectra of organic samples of the transfer set (raw spectra in reflectance, cm^−1^) **b.** 71 averaged FOSS NIRSystem5000 spectra of organic samples of the transfer set (raw spectra in absorbance, nm) **c.** 53 averaged ThermoScientific ANTARIS II spectra of organic samples of the transfer set (raw spectra in absorbance, cm^−1^) **d.** 92 averaged BUCHI NIRFlex N-500 spectra of organic samples of the transfer set (converted spectra in reflectance, nm) **e.** 53 averaged ThermoScientific ANTARIS II spectra of organic samples of the transfer set (converted spectra in absorbance, nm).

In order to build and validate transfer models, the dataset was split into two sets of spectra. A first set of 92 organic samples, listed in Appendix 1, was selected to cover a wide range of variation for cell wall composition to promote a wide range of spectra, in order to create spectra transfer models. First, spectra were acquired on the BUCHI NIRFlex N-500 device, in triplicate and then averaged (Fig. 1a). Secondly, sample spectra were acquired on the FOSS NIRSystem5000 spectrometer in duplicate and were automatically averaged (Fig. 1b). Finally, spectra were acquired on the ThermoScientific ANTARIS II device ten times and then averaged (Fig. 1c). Fig. 1d and e represents BUCHI NIRFlex N-500 spectra and ThermoScientific ANTARIS II spectra converted in nm as usually represented in the NIR field.

Spectrometer acquisition modalities were specific to each equipment: absorbance spectra in wavelengths (nm) for FOSS spectrometer, reflectance spectra in wave numbers (cm^−1^) for BUCHI spectrometer and absorbance spectra in wave numbers (cm^−1^) for ANTARIS spectrometer. Six spectra transfer models were created based on this first dataset with Piecewise Direct Standardization (PDS) [1]: transfer model from BUCHI to FOSS and vice versa, transfer model from BUCHI to ANTARIS and vice versa and transfer model from FOSS to ANTARIS and vice versa. Spectrum treatments and models were created on ChemFlow (https://vm-chemflow-francegrille.eu/) [2], a free online chemometric software that includes all the necessary functions.

**Fig. 2:**
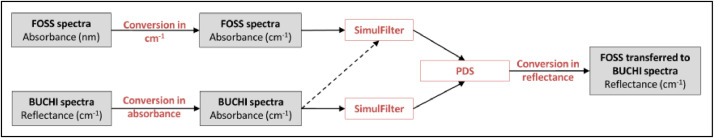
Schematic representation of the transfer model from a FOSS spectrum to a BUCHI spectrum. SimulFilter: algorithm for interpolations, PDS: Piecewise Direct Standardization.

Fig. 2 is a schematic representation of the main functions used for the transfer model from a FOSS spectrum to a BUCHI spectrum. The resulting spectra transfer model is a transform matrix (Appendix 2), allowing transforming FOSS spectra into BUCHI spectra by matrix multiplication. The other five transfer models were established in a same way and transform matrices were used alike (data not shown in the article but available in the repository).

**Fig. 3:**
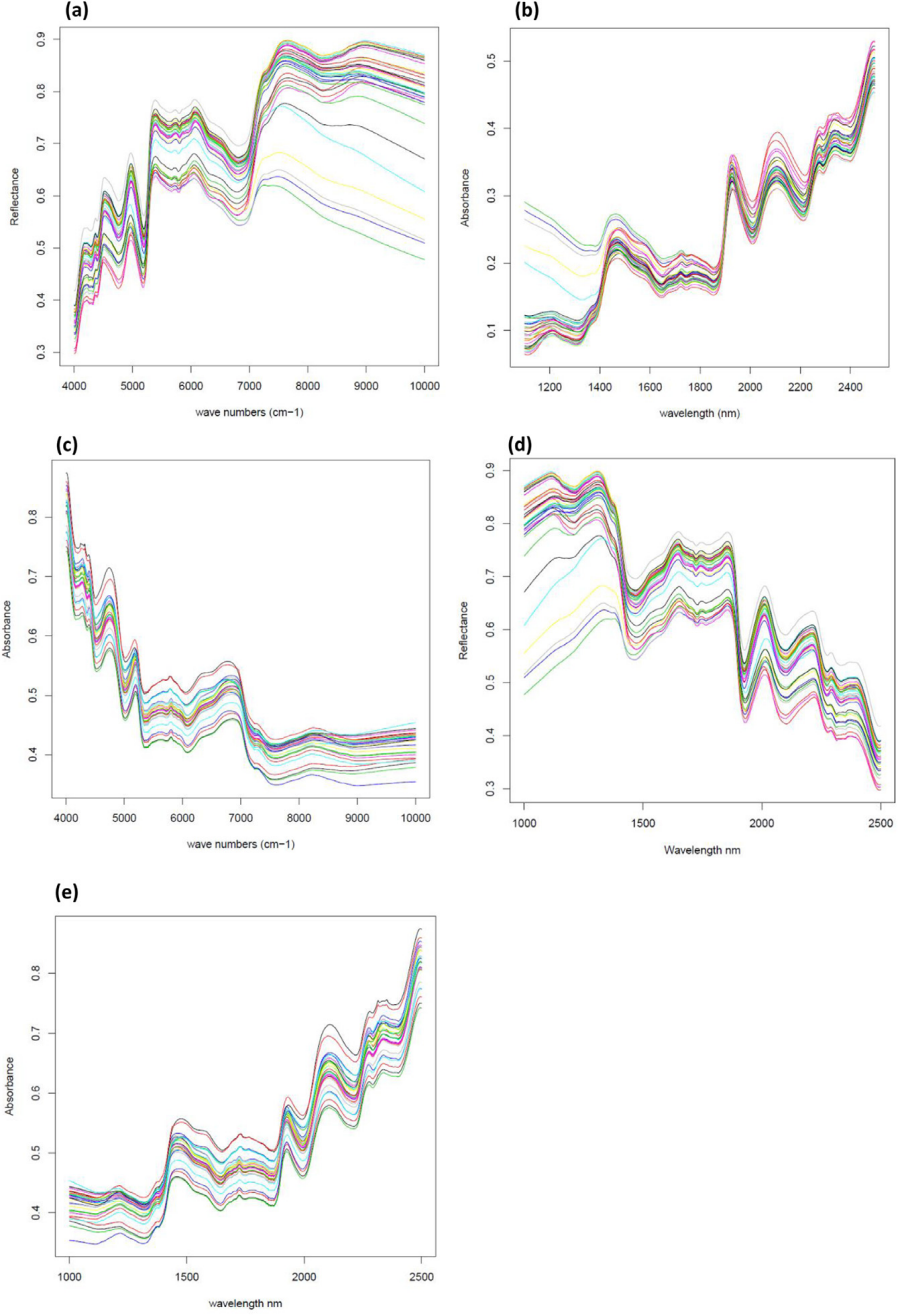
**a.** 35 averaged BUCHI NIRFlex N-500 spectra of organic samples of the transfer validation set (raw spectra in reflectance, cm^−1^) **b.** 35 averaged FOSS NIRSystem5000 spectra of organic samples of the transfer validation set (raw spectra in absorbance, nm) **c.** 29 averaged ThermoScientific ANTARIS II spectra of organic samples of the transfer validation set (raw spectra in absorbance, cm^−1^) **d.** 35 averaged BUCHI NIRFlex N-500 spectra of organic samples of the transfer validation set (converted spectra in reflectance, nm) **e.** 29 averaged ThermoScientific ANTARIS II spectra of organic samples of the transfer validation set (converted spectra in absorbance, nm).

Then, a second set, of 11 sorghum and 24 miscanthus samples, listed in Appendix 3, was selected to validate transfer models and estimate the quality of these transfers. Sample spectra of validation set were acquired on the three devices with the same modalities as before (Fig. 3a–e) and transferred with previous transfer models (data not shown in the article but available in the repository).

## 2 Experimental design, materials, and methods

### 2.1 Sample preparation

The 127 raw samples were dried either by freeze-drying or by oven drying and then ground to 1 mm size before being packaged in hermetic bottles or bags. All samples were either shared between laboratories quickly so that the spectra could be acquired on similar conditions (no material degradation) or shared with a longer time frame.

### 2.2 Data collection

FOSS spectrometer NIRSystem5000 gave absorbance spectra in wavelength with a range of 1100 nm to 2498 nm and a resolution of 2 nm. Each spectrum was an average of 32 spectral readings of the same cup. All of the 106 (71+35) samples were poured into 3.5 cm diameter cups (about 4 g) and spectra were acquired in duplicate and automatically averaged. The output data format was .csv, compatible with ChemFlow, so data were directly imported into the software and converted in .tsv format.

BUCHI N-500 spectrometer gave reflectance spectra in wave numbers, with a range of 4000 cm^−1^ to 10,000 cm^−1^ and with a resolution of 4 cm^−1^. Each spectrum was an average of 96 spectral readings of the same cup or vial. All of the 127 (92+35) samples were poured either into 10 cm diameter cups (about 30 g) or into 1.3 cm diameter vials (about 3 g) and spectra were acquired in triplicate and then averaged.

ThermoScientific ANTARIS II spectrometer gave absorbance spectra in wave numbers with a range of 4000 cm^−1^ to 10,001 cm^−1^ and with a resolution of 3.86 cm^−1^. All of the 82 (53+29) samples were divided into 3 or 10 subsamples (aliquots) and poured into 0.8 cm diameter vials (about 2 ml) which was scanned once. For each subsample, an average (spectrum) of 64 spectral readings was recorded.

The output data format for BUCHI and ANTARIS spectrometers was .jdx, compatible with ChemFlow, so data were directly imported into the software and converted in .tsv format.

### 2.3 Transfer model creation

In order to create FOSS to BUCHI spectra transfer model, spectra of the transfer set needed pretreatments to match the spectral modalities. To use the PDS function, the spectral modalities used were absorbance and cm^−1^ unit. Therefore, the first step was to transform BUCHI spectra in absorbance with the logarithm function and FOSS spectra in cm^−1^ unit with a simple unit conversion. Then, as spectra did not have the same unit values, a SimulFilter function (extrapolation function with unit width of 2) was applied to match FOSS spectra wave numbers with BUCHI spectra values. The transfer model was created with the PDS function, with a number of channels in each window equal to 7. Finally, FOSS output spectra were transformed in reflectance spectra with power function (10^−^*^x^*) to fit with BUCHI spectral modalities.

## Declaration of Competing Interest

The authors declare that they have no known competing financial interests or personal relationships which have, or could be perceived to have, influenced the work reported in this article.

## Appendix

Appendix 1 is available at this link: https://doi.org/10.15454/XFTLV4/GCPQ0Z

Appendix 2 is available at this link: https://doi.org/10.15454/XFTLV4/ZMTH60

Appendix 3 is available at this link: https://doi.org/10.15454/XFTLV4/89FTMC

## Appendix B Supplementary materials

Image, application 1

## References

[1] Wang Y., Veltkamp D.J., Kowalski B.R. (1991). Multivariate instrument standardization. Anal. Chem..

[2] Rossard V., Boulet J.C., Gogé F., Latrille E., Roger J.M. (2016). ChemFlow, chemometrics using Galaxy. Proceedings of the Galaxy Community Conference - GCC2016.

